# Longitudinal course and predictors of health-related quality of life, mental health, and fatigue, in non-hospitalized individuals with or without post COVID-19 syndrome

**DOI:** 10.1186/s12955-024-02245-y

**Published:** 2024-04-14

**Authors:** Inge Kirchberger, Christine Meisinger, Tobias D. Warm, Alexander Hyhlik-Dürr, Jakob Linseisen, Yvonne Goßlau

**Affiliations:** 1 Epidemiology, Faculty of Medicine, University of Augsburg, University Hospital of Augsburg, Stenglinstraße 2, Augsburg, Germany; 2 Vascular Surgery, Faculty of Medicine, University of Augsburg, Augsburg, Germany; 3 Institute for Medical Information Processing, Biometry and Epidemiology - IBE, LMU Munich, Munich, Germany

**Keywords:** COVID-19, Post COVID-19 syndrome, Long COVID, Mental health, Health-related quality of life, Depression, Fatigue

## Abstract

**Background:**

Long-term information on health-related quality of life (HRQOL) and mental health of non-hospitalized individuals with „post COVID-19 syndrome“ (PCS) is scarce. Thus, the objectives of the present study were to compare HRQOL and mental health of individuals with and without PCS in a German sample of non-hospitalized persons after SARS-CoV-2 infection, to characterize the long-term course up to 2 years and to identify predictors for post COVID-19 impairments.

**Methods:**

Individuals with past SARS-CoV-2 infection were examined at the University Hospital of Augsburg from November 2020 to May 2021 and completed a postal questionnaire between June and November 2022. Participants who self-reported the presence of fatigue, dyspnea on exertion, memory problems or concentration problems were classified as having PCS. HRQOL was assessed using the Veterans RAND 12-Item Health Survey, mental health was measured by the Patient Health Questionnaire and the Fatigue Asessment Scale was used to assess fatigue severity. Multivariable linear regression models with inverse probability weighting were used to determine the association between PCS and health outcomes.

**Results:**

From the 304 participants (58.2% women, median age 52 years), 210 (69.1%) were classified as having PCS in median 26 months after SARS-CoV-2 infection. Persons with PCS showed significantly more often depressive and anxiety disorders. PCS was independently and significantly associated with higher levels of depression, post-traumatic stress and fatigue, as well as poorer physical and mental HRQOL in median 9 months as well as 26 months after SARS-CoV-2 infection. A large number of acute symptoms and a prior diagnosis of depression were independently associated with poor mental health and HRQOL. While post-traumatic stress and mental HRQOL improved from 9 months to 26 months post infection onset, depressiveness, fatigue and physical HRQOL remained stable in both, persons with and without PCS.

**Conclusions:**

PCS in non-hospitalized persons after SARS-CoV-2 infection is often associated with long-term impairments of mental health and HRQOL outcomes.

## Background

A considerable proportion of patients infected with the coronavirus SARS-CoV-2 reports persisting symptoms such as fatigue, dyspnea, and cognitive problems, for weeks or months after the acute coronavirus disease 2019 (COVID-19) [1–4]. This long-term sequelae is commonly called „long COVID“ or „post COVID syndrome/condition“ [5, 6]. The prevalence of post COVID syndrome (PCS), which includes persistence of symptoms for at least 12 weeks, varies depending on the specific definition of PCS, the study design and symptom assessment, and the severity of the acute COVID-19, and ranges between 6% and 46% in non-hospitalized persons [3, 4, 7–9]. The prevalance in non-hospitalized persons is particularly important, since this group makes up 80% [10] to 97% [8] of all COVID-19 cases. Since many of the persons with PCS may require healthcare, a large number of affected individuals would challenge the healthcare systems [11].

Overall, the number and range of single persisting symptoms after SARS-CoV-2 infection have been investigated in several studies [12], but patient-reported outcomes measures (PROMS) which enable a comprehensive assessment of the possible effects of persisting symptoms on an individuals‘ mental health and HRQOL were only rarely included in studies so far [13–14].

Available results on prevalences of mental health problems in individuals with PCS show a wide range depending on study population and– design [12, 15]. For instance, depression or depressive symptoms were found in 11 to 28% (depressive symptoms + 12 weeks following SARS-CoV-2 infection) [16] and 81.5% (at least mild depressive symptoms in median 163 days following SARS-CoV-2 infection) [17]. Similarly, metaanalyses showed that 1 to 17% of the persons with COVID-19 developed a posttraumatic stress disorder (PTSD) [14, 18], whereas other studies reported prevalences of PTSD around 30% in individuals with PCS [19, 20]. In terms of HRQOL, a metaanalysis of twelve studies found a pooled prevalence of impaired HRQOL (EQ-5D VAS) in 59% of the persons with PCS [21].

Available study results are often based on mixed samples of hospitalized and non-hospitalized persons and investigations on the persistence of health limitations with follow-up times exceeding one year are lacking so far. Moreover, predictors of long-term impairments of mental health and HRQOL are not comprehensively determined. However, long-term information is needed to assure that healthcare services appropriately consider specific short- and longterm needs of individuals with PCS.

Thus, the objectives of the present study were to compare HRQL and mental health of individuals with and without PCS in a German sample of non-hospitalized persons after SARS-CoV-2 infection, to characterize the long-term course up to 2 years and to identify predictors of post COVID-19 impairments.

## Methods

### Design and study population

**Fig. 1:**
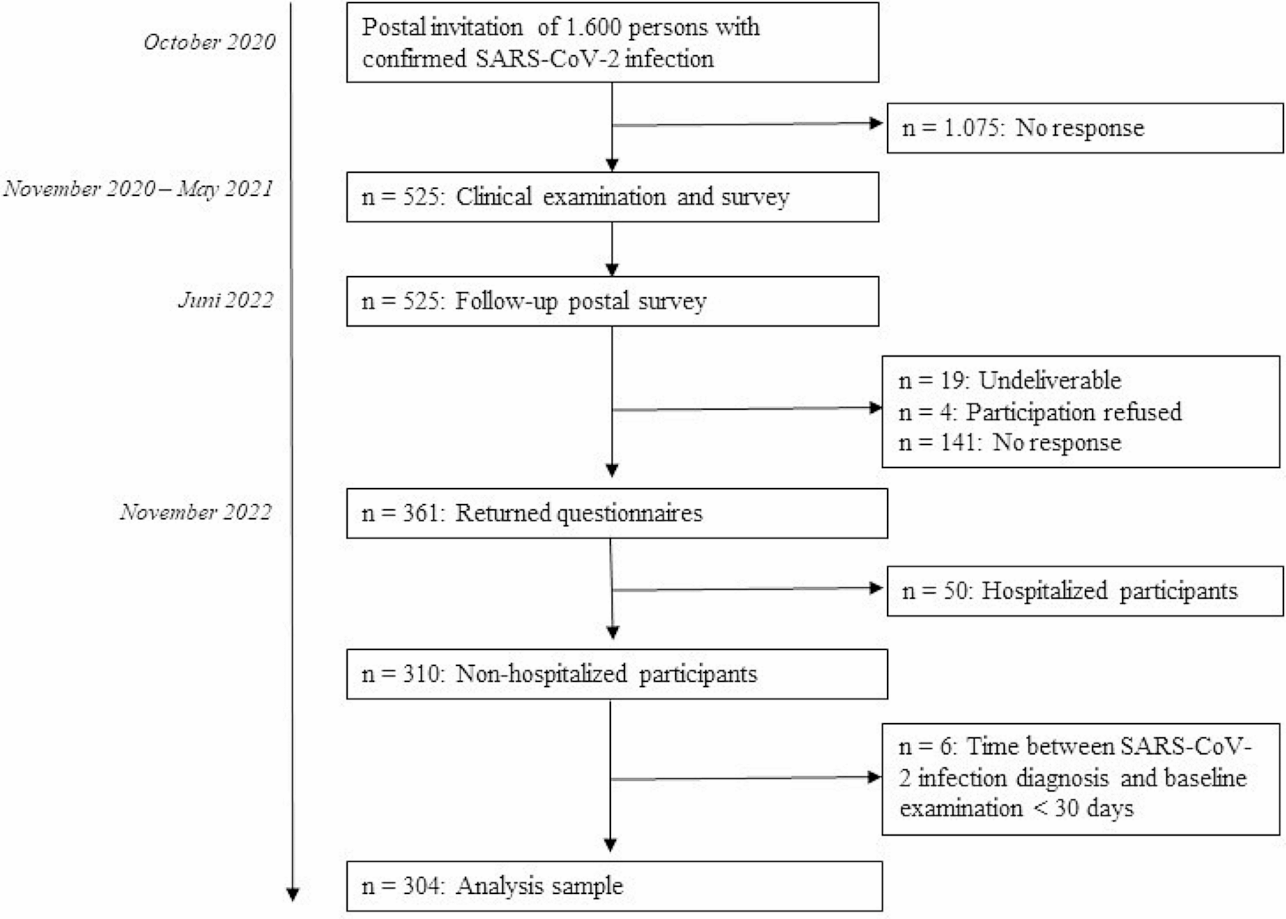
Flow chart

The present study is a follow-up assessment of the Corona Thrombosis Study (COVID-T), a prospective single-center observational study evaluating the consequences of SARS-CoV-2 infection on the vascular system [22–24]. The study sample was recruited from the population living in the city and the county of Augsburg. The public health departments identified eligible persons with past SARS-CoV-2 infection confirmed by positive polymerase chain reaction (PCR) testing and sent out a total of 1600 postal invitations for study participation between 21 October 2020 and 6 November 2020. The potential study participants were invited for clinical examinations and assessments that were performed at the University Hospital of Augsburg from 4 November 2020 to 26 May 2021. From the 1600 invited persons, a total of 525 (32.8%) participants were enrolled in the study. A postal follow-up survey was conducted between 14 Juni 2022 and 1 November 2022. From the 525 persons, 361 (69%) returned a completed questionnaire. The present analysis is based on 304 persons who were not hospitalized for their initial SARS-CoV-2 infection (see Fig. 1).

The study was approved by the ethics committee of the Ludwig-Maximilians Universität Munich and was performed in accordance with the Declaration of Helsinki. Written informed consent was obtained from all participants.

### Measures

Data was collected using a self-reporting questionnaire which was administered on a tablet personal computer at the baseline examination and on paper at the postal follow-up survey. The questionnaire covered information on socio-demographics, disease history, comorbid conditions (collected at baseline) as well as symptoms during the acute SARS-CoV-2 infection and persisting symptoms. The participants were asked to complete a self-developed list of 42 symptoms, rating them for their occurrence in the acute phase as well as for the 14 days before the the baseline examination and the follow-up survey.

Moreover, standardized questionnaires were used to assess depression and anxiety, PTSD, fatigue and HRQOL at both measure points. The German version of the Patient Health Questionnaire (PHQ-D) was applied to determine the frequency of suspected depressive and anxiety disorders and the extent of depressiveness [25–27]. Depressive symptoms were assessed with the depression module of the Patient Health Questionnaire (PHQ-9). The PHQ-9 consists of a sum of nine items scoring with a range from 0 to 27. A score less than five can be interpreted as the absence of depressiveness. Values between 5 and 10 constitute a mild degree of depressiveness. Values of 10 and higher can be subdivided into moderate (10 to 14), moderately severe (15 to 19), and severe (20 to 27) depressiveness [25]. The German version of the PHQ-9 showed good psychometric properties [28].

PTSD was measured using the revised Impact of Event Scale (IES-R). The IES-R consists of three subscales made up of 22 items. Based on a weighted summation of the subscale scores, a total score can be calculated which indicates the likelihood of a suspected diagnosis of PTSD. A score of 0 or below indicates no suspected diagnosis of PTSD, whereas scores above 0 suggest a suspected PTSD diagnosis [29].

Fatigue was assessed using the Fatigue Assessment Scale (FAS), which was shown to be a reliable and valid questionnaire [30, 31]. The FAS consists of10 items which make up a summary score with a minimum of 10 and a maximum of 50. Scores below 24 indicate no fatigue. Persons scoring between 24 and 35 can be classified as having moderate fatigue and individuals with scores above 35 show a high level of fatigue [32].

HRQOL was assessed using the Veterans RAND 12-Item Health Survey (VR-12). The VR-12 consists of 12 items which make up two subscales, physical and mental HRQOL. The scores range between 0 and 100 with higher values indicating better HRQOL [33].

### Definition of post COVID-19 syndrome

In the present study, a definition of the PCS largely based on the WHO clinical case definition [5] was applied. This PCS definition suggests fatigue, shortness of breath and cognitive dysfunction as the most important symptoms of PCS amongst a number of additional symptoms. Thus, the classification of PCS in the current study is based on the participants responses to the questions whether they had experienced fatigue or exhaustion, dyspnea on exertion, memory problems or concentration problems during the past 14 days before the survey. Response options were „yes“ or „no“. Participants who reported at least one of these 4 symptoms either at the baseline assessment (median 9 months after acute infection) or at follow-up (median 26 months after acute infection), were classified as having PCS.

### Data analysis

Chi square test or Fisher’s exact test were used to determine differences between persons with or without PCS in nominal variables and Mann-Whitney U-Test in continuous variables without normal distribution, respectively. Wilcoxon test was applied to test for changes between baseline and follow-up (FUP) assessments. Effect sizes (r) were derived from z-scores divided by the rooted sample size. According to Cohen [34] r values between 0.1 and 0.3 are small effects, 0.3–0.5 are moderate effects, and 0.5 and higher are large effects.

Multivariable linear regression models adjusted for relevant confounders were calculated to estimate the association between PCS and continuous health outcomes. Relevent confounding variables were selected based on the available literature. In order to adjust for a bias due to loss to follow-up, inverse probability weighting was applied. The assumptions for multivariable linear regression analyses were tested using scatterplots and Q-Q plots to confirm linearity of associations, normal distribution of the residuals and homoscedasticity. Cook’s distances and leverage diagnostic plots were used to check for leveraging outliers. Variance inflation factors were used to identify multicollinearity and Durbin Watson tests were calculated to determine autocorrelation. For statistical tests an alpha level of 0.05 was defined. Statistical analyses were performed using SAS Version 9.4.

## Results

### Sample characteristics

The study sample consisted of 177 (58.2%) women and 127 (41.8%) men with a median age of 52 years. From the 304 participants, 154 (50.7%) had PCS at baseline, 187 (61.5%) at FUP, 23 (7.6%) persons had PCS at baseline but not at FUP, and 56 (18.4%) had PCS at FUP but not at baseline. According to the definition applied in the present study, 210 (69.1%) were classified as having PCS either at baseline or FUP. Further characteristics are detailed in Table 1. The proportion of participants with a BMI > 30 kg/m² and with a prior diagnosis of depression was significantly larger in participants with PCS.

Compared with the 142 persons not responding to the follow-up survey, the responding participants were significantly less often male or living alone, had more post-COVID symptoms and were older.

**Table 1:** Sample characteristics

	Total (*n* = 304)		PCS yes (*n* = 210)		PCS no (*n* = 94)		
	n	%	n	%	n	%	*p*-value
Sex							0.0904^1^
Male	127	41.78	81	38.57	46	48.94	
Female	177	58.22	129	61.43	48	51.06	
Age							
Median (IQR)	52	(40;59)	52	(40;59)	52	(39;60)	0.3455^2^
Mean (SD)	49.41	(14.52)	48.92	(14.57)	50.49	(14.45)	
Education							0.6278^1^
<= 9 years	52	17.10	40	19.05	18	19.15	
> 9 years	252	82.89	170	80.95	76	80.85	
Living alone, yes	69	22.70	45	21.74	24	25.81	0.4388^1^
Smoking							0.2792^1^
Never smoker	160	52.63	108	51.43	52	55.32	
Ex-smoker	123	40.46	90	42.86	33	35.11	
Current smoker	21	6.91	12	5.71	9	9.57	
Body Mass Index							0.0306^1^
<= 30 kg/m²	254	83.55	169	80.48	85	90.43	
> 30 kg/m²	50	16.45	41	19.52	9	9.57	
Comorbidities							
Hypertension	64	21.12	41	19.62	23	24.47	0.3386^1^
Diabetes	14	4.62	11	5.26	3	3.19	0.5611^1^
Myocardial infarction	7	2.31	6	2.87	1	1.06	0.3328^1^
Coronary artery disease	16	5.28	14	6.70	2	2.13	0.1622^1^
Stroke	6	1.98	4	1.91	2	2.13	1.0000^1^
Anxiety disorder	18	5.94	15	7.18	3	3.19	0.0560^1^
Chronic bronchitis	19	6.29	17	8.17	2	2.13	0.0771^1^
Depression	27	8.88	23	10.95	4	4.26	0.0280^1^
Autoimmune disorder	28	9.24	20	9.57	8	8.51	0.8877^1^
Cancer	15	4.93	10	4.76	5	5.32	0.6259^1^
Recurrent COVID-19	73	24.58	54	26.47	19	20.43	0.5332^1^
Time between first positive PCR test and follow-up survey							0.6679^3^
> 12 to < = 15 months	8	2.63	5	2.38	3	3.19	
> 15 bis < = 18 months	13	4.28	10	4.76	3	3.19	
> 18 bis < = 21 months	74	24.34	56	26.67	18	19.15	
> 21 bis < = 24 months	52	17.11	32	15.24	20	21.28	
> 24 bis < = 27 months	62	20.39	43	20.48	19	20.21	
> 27 bis < = 30 months	94	30.92	63	30.00	31	32.98	
> 30 months	1	0.33	1	0.48	0	0	
Median (IQR)	26	(20.5;27.2)	25.91	(20.15;27.16)	26.02	(20.71;27.16)	0.5134^2^
Mean (SD)	23.75	(3.92)	23.66	(3.97)	23.96	(3.80)	
Min/Max		14.1/30.2		14.1/30.2		14.20/29.82	

PCS: Post Covid-19 syndrome

^1^Chi-Square test; ^2^Mann-Whitney U-test; ^3^Fisher’s exact test

Among the symptoms which were part of the PCS classification, fatigue was the most common symptom at baseline (*n* = 103, 33.9%) and FUP (*n* = 158, 52.8%), followed by concentration problems (*n* = 82, 27.2%, *n* = 105, 34.5%) and memory problems (*n* = 71, 23.4%, *n* = 101, 33.2%). Dyspnea on exertion was reported by 74 participants (24.4%) at baseline and 82 participants (27.0%) at FUP. In addition, the median number of symptoms at baseline was 6 (3;10) in persons with PCS and 1 (0;2) in persons without PCS. At FUP persons with PCS had a median of 9 (5;15) symptoms compared with persons without PCS who had a median of 1 (0;3) symptom. Differences at both time points were significant (*p* < 0.0001).

### Health outcomes

According to the PHQ-D, none of the participants without PCS had an indication of a depressive or anxiety disorder, whereas 3 to almost 10% of the participants with PCS had scores indicating the presence of such a disease (see Table 2). A significantly higher proportion of affected participants at FUP compared with baseline was found for major depression (*p* < 0.0001), other depressive syndromes (*p* = 0.0010) and other anxiety syndromes (*p* = 0.0016). No significant change from baseline to FUP was found for panic syndromes (*p* = 0.1944).

**Table 2:** Indication of depressive and anxiety disorders according to the PHQ-D

		Total (*n* = 304)		PCS yes (*n* = 210)		PCS no (*n* = 94)		
		n	%	n	%	n	%	*p*-value^1^
**Baseline**								
Major depression		11	3.65	11	5.31	0	0	0.0198
Other depressive syndrome		8	2.66	8	3.86	0	0	0.0608
Panic syndrome		7	2.33	7	3.35	0	0	0.1027
Other anxiety syndrome		7	2.31	7	3.35	0	0	0.1036
**Follow-up**								
Major depression		20	6.58	20	9.57	0	0	0.0007
Other depressive syndrome		10	3.29	10	4.78	0	0	0.0344
Panic syndrome		9	2.97	9	4.31	0	0	0.0614
Other anxiety syndrome		12	3.99	12	5.71	0	0	0.0209

PCS: Post Covid-19 syndrome

^1^Fisher’s exact test

Table 3 shows that participants with PCS had significantly more posttraumatic stress, depressiveness, and fatigue, as well as worse mental and physical HRQOL at both measure points. This significant association remained after adjusting for potential confounders (see Table 4). The number of symptoms in the acute phase was a further significant predictor of all outcomes. History of anxiety or depression disorders showed consistently significant associations with depressiveness and mental HRQOL.

**Table 3:** Differences in health outcomes between participants with and without PCS

	Total (*n* = 304)		PCS yes (*n* = 210)		PCS no (*n* = 94)			
							*p*-value^1^	Effect size r
**Baseline**								
Postraumatic stress (IES-R )								
Median (IQR)	-3.64	(-4.21;-2.61)	-3.38	(-4.07;-2.13)	-4.08	(-4.36;-3.46)	< 0.0001	0.29
Mean (SD)	-3.20	(1.30)	-2.97	(1.37)	-3.70	(0.97)		
Depressiveness (PHQ-9)								
Median (IQR)	4	(2;7)	5	(3;9)	2	(1;3)	< 0.0001	0.46
Mean (SD)	4.68	(3.98)	5.78	(4.08)	2.24	(2.34)		
Fatigue (FAS)								
Median (IQR)	19	(15;24)	21	(17;27)	15	(13;18)	< 0.0001	0.51
Mean (SD)	20.67	(7.49)	22.96	(7.62)	15.56	(3.75)		
Physical HRQOL (VR-12)								
Median (IQR)	51.77	(44.28;55.52)	48.46	(42.02;54.66)	54.81	(52.74; 55.88)	< 0.0001	0.38
Mean (SD)	48.96	(8.60)	46.95	(9.06)	53.45	(5.22)		
Mental HRQOL (VR-12 )								
Median (IQR)	50.74	(44.32;56.33)	48.71	(41.25;54.14)	55.64	(51.19;59.30)	< 0.0001	0.39
Mean (SD)	49.53	(9.05)	47.29	(9.21)	54.53	(6.30)		
**Follow-up**								
Postraumatic stress (IES-R )								
Median (IQR)	-3.95	(-4.36;-3.05)	-3.76	(-4.21;-2.53)	-4.25	(-4.36;-3.91)	< 0.0001	0.34
Mean (SD)	-3.47	(1.19)	-3.23	(1.30)	-4.01	(0.59)		
Depressiveness (PHQ-9)								
Median (IQR)	4	(2;6)	5	(3;8)	1	(0;6)	< 0.0001	0.52
Mean (SD)	4.74	(4.50)	6.09	(4.71)	1.74	(1.72)		
Fatigue (FAS)								
Median (IQR)	20	(15;25)	22	(17;29)	14.5	(12;18)	< 0.0001	0.51
Mean (SD)	21.15	(8.43)	23.77	(8.61)	15.30	(3.89)		
Physical HRQOL (VR-12)								
Median (IQR)	51.32	(43.60;54.81)	48.48	(40.61;53.49)	54.76	(51.56;55.66)	< 0.0001	0.36
Mean (SD)	48.37	(8.33)	46.53	(8.69)	52.48	(5.65)		
Mental HRQOL (VR-12 )								
Median (IQR)	54.43	(46.39;59.24)	51.26	(42.06;56.56)	58.53	(54.94;59.34)	< 0.0001	0.40
Mean (SD)	51.06	(10.20)	48.50	(10.91)	56.80	(4.83)		

PCS: Post Covid-19 syndrome; IQR: Interquartile range; SD: Standard deviation; IES-R: Revised Impact of Event Scale; PHQ-9: Patient Health Questionnaire; FAS: Fatigue Assessment Scale; VR-12: Veterans RAND 12-Item Health Survey

^1^Mann-Whitney U-test

**Table 4:** Association of PCS with health outcomes: multivariable linear regression models with inverse probability weighting

	Baseline			Follow-Up		
	ß	95% CI	*p*-value	ß	95% CI	*p*-value
**Depression (PHQ-9)**						
PCS (yes)	2.20	1.36; 3.04	< 0.0001	3.06	2.10; 4.01	< 0.0001
Gender (female)	0.63	-0.12; 1.38	0.0985	0.27	-0.57; 1.12	0.5260
Age (years)	0.01	-0.02; 0.03	0.7320	-0.03	-0.06; -0.01	0.0530
School education (less than 10 years)	0.62	-0.42; 1.65	0.2399	0.70	-0.47; 1.88	0.2410
Living alone (yes)	-0.68	-1.56; 0.19	0.1256	-1.00	-1.98; -0.02	0.0462
History of anxiety disorder (yes)	2.81	0.98; 4.64	0.0028	3.62	1.53; 5.71	0.0007
History of depressive disorder (yes)	2.25	0.78; 3.71	0.0028	2.23	0.56; 3.89	0.0091
Sum of acute symptoms	0.17	0.11; 0.22	< 0.0001	0.17	0.11; 0.23	< 0.0001
**Fatigue (FAS)**						
PCS (yes)	4.63	3.09; 6.17	< 0.0001	5.92	4.13; 7.71	< 0.0001
Gender (female)	1.38	-0.01; 2.76	0.0492	1.43	-0.17; 3.02	0.0790
Age (years)	-0.02	-0.07; 0.03	0.4334	-0.02	-0.08; 0.04	0.4995
School education (less than 10 years)	1.57	-0.33; 3.47	0.1041	1.47	-0.74; 3.68	0.1913
Living alone (yes)	-0.13	-1.72; 1.46	0.8711	-0.45	-2.29; 1.39	0.6281
History of anxiety disorder (yes)	3.98	0.61; 7.36	0.0210	3.72	-0.21; 7.65	0.0631
History of depressive disorder (yes)	2.08	-0.61; 4.78	0.1292	3.39	0.25; 6.52	0.0344
Sum of acute symptoms	0.37	0.28; 0.47	< 0.0001	0.29	0.18; 0.40	< 0.0001
**Posttraumatic stress (IES-R)**						
PCS (yes)	0.28	-0.00; 0.56	0.0531	0.40	0.14; 0.66	0.0031
Gender (female)	0.34	0.09; 0.59	0.0077	0.11	-0.13; 0.34	0.3691
Age (years)	0.01	0.00; 0.02	0.0542	0.01	-0.01; 0.01	0.4804
School education (less than 10 years)	0.32	-0.03; 0.66	0.0734	0.59	0.27; 0.91	0.0004
Living alone (yes)	-0.45	-0.74; -0.16	0.0027	-0.21	-0.48; 0.06	0.1326
History of anxiety disorder (yes)	0.24	-0.38; 0.86	0.4416	0.42	-0.16; 0.99	0.1531
History of depressive disorder (yes)	0.26	-0.24; 0.75	0.3075	0.54	0.08; 0.99	0.0211
Sum of acute symptoms	0.07	0.05; 0.08	< 0.0001	0.05	0.04; 0.07	< 0.0001
**Physical quality of life (VR-12)**						
PCS (yes)	-4.04	-5.80; -2.28	< 0.0001	-3.54	-5.29; -1.80	< 0.0001
Gender (female)	-1.78	-3.35; -0.21	0.0260	-3.22	-4.77; -1.67	< 0.0001
Age (years)	-0.16	-0.22; -0.11	< 0.0001	-0.14	-0.20; -0.09	< 0.0001
School education (less than 10 years)	-1.99	-4.16; 0.18	0.0714	-2.39	-4.53; -0.24	0.0292
Living alone (yes)	0.39	-1.42; 2.19	0.6744	0.61	-1.18; 2.40	0.5042
History of anxiety disorder (yes)	-0.27	-4.13; 3.59	0.8894	-1.42	-5.24; 2.39	0.4641
History of depressive disorder (yes)	0.33	-2.75; 3.41	0.8347	0.10	-2.95; 3.14	0.9498
Sum of acute symptoms	-0.42	-0.53; -0.31	< 0.0001	-0.34	-0.44; -0.23	< 0.0001
**Mental quality of life (VR-12)**						
PCS (yes)	-4.18	-6.16; -2.19	< 0.0001	-5.30	-7.48; -3.12	< 0.0001
Gender (female)	-2.55	-4.31; -0.78	0.0048	-1.20	-3.14; 0.74	0.2250
Age (years)	0.04	-0.02; 0.11	0.1759	0.09	0.02; 0.16	0.0098
School education (less than 10 years)	-1.87	-4.31; 0.57	0.1330	-2.37	-5.05; 0.32	0.0841
Living alone (yes)	1.65	-0.39; 3.68	0.1124	2.34	0.10; 4.57	0.0410
History of anxiety disorder (yes)	-4.47	-8.82; -0.13	0.0438	-7.66	-12.44; -2.89	0.0018
History of depressive disorder (yes)	-6.83	-10.30; -3.36	0.0001	-5.88	-9.69; -2.06	0.0027
Sum of acute symptoms	-0.29	-0.41; -0.17	< 0.0001	-0.30	-0.43; -0.17	< 0.0001

PCS: Post Covid-19 syndrome; IES-R: Revised Impact of Event Scale; PHQ-9: Patient Health Questionnaire; FAS: Fatigue Assessment Scale; VR-12: Veterans RAND 12-Item Health Survey

A significant improvement of post-traumatic stress symptoms over time were found in patients with and without PCS (see Table 5). Similarly, mental HRQOL significantly improved in both groups. Effect sizes were larger in participants without PCS. No significant changes were found regarding depressiveness, fatigue and physical HRQOL.

**Table 5:** Change of health outcomes from baseline to follow-up

	Total (*n* = 304)				PCS yes (*n* = 210)				PCS no (*n* = 94)			
			*p*-value^1^	Effect size r			*p*-value^1^	Effect size r			*p*-value^1^	Effect size r
Postraumatic stress (IES-R )												
Median (IQR)	-0.13	(-0.73;0.17)	< 0.0001	0.22	-0.13	(-0.75; 0.31)	0.0016	0.20	-0.14	(-0.63; 0.01)	< 0.0001	0.29
Mean (SD)	-0.25	(1.01)			-0.23	(1.08)			-0.30	(0.83)		
Depressiveness (PHQ-9)												
Median (IQR)	0.0	(-2.0; 2.0)	0.9102	< 0.01	0	(-2; 2)	0.3796	0.05	0	(-1; 1)	0.0514	0.11
Mean (SD)	0.05	(3.22)			0.31	(3.60)			-0.50	(2.07)		
Fatigue (FAS)												
Median (IQR)	0	(-3; 4)	0.2472	0.06	1	(-3; 5)	0.0860	0.11	0	(-3; 1)	0.3018	0.08
Mean (SD)	0.45	(6.24)			0.77	(7.09)			-0.26	(3.65)		
Physical HRQOL (VR-12)												
Median (IQR)	-0.75	-4.18; 3.10	0.1165	0.09	-0.82	(-4.57; 4.12)	0.3890	0.06	-0.60	(-3.23; 1.49)	0.0839	0.15
Mean (SD)	-0.59	(7.26)			-0.42	(7.90)			-0.96	(5.59)		
Mental HRQOL (VR-12 )												
Median (IQR)	1.23	(-3.55; 6.76)	0.0006	0.19	1.28	(-5.62; 7.72)	0.0407	0.14	0.87	(-0.90; 4.74)	0.0004	0.30
Mean (SD)	1.53	(8.95)			1.20	(10.12)			2.27	(5.48)		

PCS: Post Covid-19 syndrome; IES-R: Revised Impact of Event Scale; PHQ-9: Patient Health Questionnaire; FAS: Fatigue Assessment Scale; VR-12: Veterans RAND 12-Item Health Survey

^1^ Wilcoxon test

## Discussion

The present study found that even 2 years after SARS-CoV-2 infection 69.1% of non-hospitalized persons were classified as having PCS. Persons with PCS showed significantly more often depressive and anxiety disorders, higher levels of depressiveness, post-traumatic stress and fatigue, as well as poorer physical and mental HRQOL compared with persons without PCS. While post-traumatic stress and mental HRQOL improved from 9 months to 26 months post infection onset, depressiveness, fatigue and physical HRQOL remained stable in both, persons with and without PCS. The proportion of individuals with PCS with suspected depressive and anxiety disorders even increased over time. Besides PCS, the number of acute symptoms and a prior diagnosis of depression were independently associated with mental health and HRQOL outomes.

Overall, the results of the present study confirmed that PCS considerably affects mental health, fatigue and HRQOL even 2 years after the acute SARS-CoV-2 infection. Comparable associations were shown in studies with shorter follow-up times and/or in hospitalized patients [34, 35]. For instance, Gaspar et al. [35] reported that PCS was a significant predictor of impairment of at least one HRQOL domain (assessed by EQ-5D-3 L) 3, 6 and 9 months after COVID-19 in hospitalized individuals. Bahmer et al. [36] showed that among 667 participants from a German Cohort study, persons with higher PCS scores had significantly more severe fatigue and more anxiety and depression 9 months after SARS-CoV-2 infection.

Of interest, the significant associations of PCS with all outcomes remained even after adjustment for a past diagnosis of a depressive or anxiety disorder. This suggests that the negative effect of PCS is not restricted to persons with a high vulnerability for poor mental health. However, history of mental diseases as well as number of acute symptoms were major risk factors of poor health outcomes 2 years post SARS-CoV-2 infection also in the present study [16, 37]. Thus, persons with a history of mental diseases or a large number of acute symptoms are specific risk groups, which may require more support by healthcare providers.

In the present study it was shown that post-traumatic stress and mental HRQOL improved from 9 months to 26 months, but depressiveness, fatigue and physical HRQOL remained stable in both, persons with and without PCS. Within the subgroup of individuals without PCS the stability of scores seems plausible because the scores at 9 months indicate no or only minor deviations from population norms [15, 38]. For instance, the subgroup of individuals without PCS had mean PHQ-9 scores of 2.24 (± 2.34) which is even lower than the mean scores observed in the German population before the SARS-CoV-2 pandemic (Mean 3.9 ± 3.7) [39]. In contrast, persons with PCS showed considerable impairments in terms of mental health and HRQOL which only partly improved over time. Alarmingly, the proportion of persons with PCS who had PHQ-D scores indicative of a major depression, other depressive syndromes and other anxiety syndromes even increased significantly from 9 to 26 months post SARS-CoV-2 infection. A considerable higher proportion of persons with PCS (9.57%) had scores indicative of a major depression, compared with 2.9% of the German population before the SARS-CoV-2 pandemic [39]. Overall, these findings are in line with some prior studies reporting a lack of improvement of an initially impaired mental health status after SARS-CoV-2 infection. For instance, Gaspar et al. [35] showed that the anxiety/depression domain from the EQ-5D-3 L remained stable from 3 to 9 months post COVID-19 in persons with PCS and an increase of symptoms of depression and anxiety from 3 to 6 months to ≥ 6 months was reported by Pemrai et al. [40]. However, these studies investigated an early post SARS-CoV-2 infection time interval, whereas the present study confirmed the persistance of mental health impairments in persons with PCS up to 2 years. A variety of causes may account for a persistance or deterioration of depression and anxiety, including individual characteristics (e.g. health literacy, coping, resilience) or environmental factors (e.g. access to mental health services). The latter one also concerns the lack of specialized health care for individuals with PCS and the dissatisfaction of affected persons with PCS care [24].

To our knowledge, this is the first study which is based on a two-year follow-up of non-hospitalized persons with SARS-CoV-2 infection investigating PCS and PROMS in Germany. Only persons with confirmed positive PCR testing were included in the study. A strength of the present study is the application of inverse probability weighting in order to control for a selection bias due to loss to follow-up. A limitation which applies to all studies investigating PCS is the lack of a common definition for long COVID and PCS. This limits the comparability of results across studies. In addition, a clinical diagnosis of post-COVID symptoms and mental conditions is missing. Also, there is a certain overlap of exposure and outcome in terms of depressive symptoms. Fatigue and concentration problems are part of the PCS definition (exposure) but also included in the PHQ-D questionnaire for the assessment of depressive conditions (outcome). Although these symptoms are neither mandatory for being classified as PCS nor for a suspected depression diagnosis according to the PHQ-D, this fact may have affected the association between PCS and depressive conditions. Furthermore, the proportion of persons with PCS and the limitations of health outcomes might be overestimated because study participants may have experienced a higher disease burden than those who rejected participation. In addition, psychosocial factors and the growing media attention to PCS might have influenced the report of symptoms [41].

## Conclusions

In conclusion, among all persons with PCS a considerable proportion suffers from persistent mental health problems which develop independently from past diagnoses of depression and anxiety disorders. The results of the present study highlight the need of an early identification of persons who are at risk of developing mental health problems, to foster early access to appropriate interventions in order to avoid a persistence or deterioration of impaired mental HRQOL and mental health disorders. Further long-term studies are required to gain comprehensive knowledge on the course of PCS and its associations with HRQOL and mental health, the perceptions and needs of the affected individuals, and how the healthcare system can meet these needs.

## Data Availability

The datasets generated and/or analysed during the current study are not publicly available due to data protection requirements but are available in an anonymized form from the corresponding author on reasonable request.
